# Problematic social media use and mental health risks among first-year Chinese undergraduates: a three-wave longitudinal study

**DOI:** 10.3389/fpsyt.2023.1237924

**Published:** 2023-09-07

**Authors:** Wanqi Zhou, Zhihao Yan, Zeyang Yang, Zaheer Hussain

**Affiliations:** ^1^Department of Psychology, School of Education, Soochow University, Suzhou, China; ^2^School of Educational Science, Anhui Normal University, Wuhu, China; ^3^School of Social Sciences, Nottingham Trent University, Nottingham, United Kingdom

**Keywords:** problematic social media use, mental health risks, first-year Chinese undergraduates, longitudinal study, cross-lagged analysis

## Abstract

**Introduction:**

The association between social media use and mental health risks has been widely investigated over the past two decades with many cross-sectional studies reporting that problematic social media use (PSMU) is associated with higher mental health risk such as anxiety and depression. The present study examined the relationship between PSMU severity and mental health risks (depression, anxiety, stress, and loneliness) using a three-wave longitudinal design.

**Methods:**

A total of 685 first-year Chinese undergraduate students (Mean age = 19.12 years, SD = 0.92) completed surveys at three times points with intervals of 3 to 4 months. Results revealed that PSMU was positively correlated with all the mental health risk variables over the three time points.

**Results:**

The prevalence of PSMU increased over the three research waves. Cross-lagged models identified bi-directional relationships between PSMU and mental health risks, while such links were not consistent between different mental health risk variables and can change over different research intervals.

**Discussion:**

This study indicates that PSMU and mental health risks could predict each other in a vicious loop, but the differences between specific mental health risks and the research context (e.g., different term times and experiences in university) should not be ignored. Further research attention should be paid to the prevalence of PSMU and mental health conditions among Chinese first-year undergraduates who appear to have difficulties in adapting to university life.

## Introduction

There are more than 5 billion internet users and 4.7 billion social media users worldwide (1, 2). In 2022, China had 983.3 million active social media users which ranked it at first place globally (1, 2). Studies have frequently pointed out that social media use, especially problematic social media use (PSMU) or social media addiction, can be associated with mental health risks such as anxiety and depression (3, 4). PSMU, often regarded as one type of specific problematic internet use, can be defined as the compulsive or excessive use of social media applications (e.g., Twitter and Facebook) with addictive characteristics (e.g., mood modification, withdrawal symptoms and conflicts) that lead to functional impairments or negative consequences (5–8). However, the relationship between social media use and mental health conditions remains debatable since some studies also suggest that social media use is not linked with mental health issues and people’s decreased well-being might not be directly caused by their social media usage (9, 10). Furthermore, researchers have warned that daily behaviours should not be overpathologized as addictions (11). Several longitudinal studies have shown that there might be bi-directional relationship between addictive internet use or smartphone use and mental health risks [e.g., anxiety and depression (12, 13)]. Anxiety, depression, stress, and loneliness are among the most significant mental health risks associated with problematic social media use (PSMU) or internet addiction (3, 4, 14, 15). For example, among 1,052 participants, Ostovar et al. (14) identified that anxiety, depression, stress, and loneliness are the four significant mental health risks associated with internet addiction in a structural equation model. Because of the important association between the four variables and problematic internet use identified in many empirical studies, the present study aimed to explore the link between PSMU and anxiety, depression, stress, and loneliness using a longitudinal design. Several recent longitudinal investigations have examined the link between problematic social media use (PSMU) and mental health risks among different populations [e.g., (6, 16, 17)]. Li et al. (18) found a bi-directional link between social media addiction and depression among adolescents in China. It remains unknown whether this link can be identified in specific groups of young people, for example, first-year undergraduates who were found to have difficulties in adapting to college life (19). Overall, it is necessary to conduct longitudinal studies to track the changes of the severity of PSMU and explore the relationship between PSMU and mental health risks, especially among the first-year Chinese undergraduates during their adaptation period in university.

## Literature review

### Problematic social media use and mental health

Earlier studies tended to use the term internet addiction to describe excessive online behaviours with negative outcomes (20). Davis (21) believed that pathological internet use should be described as generalized and specific. Problematic or addictive behaviours on specific online functions or applications such as social media, online games, online gambling, and online shopping were then widely investigated in various contexts (8, 22–24). Theoretically, the Interaction of Person-Affect-Cognition-Execution (IPACE) model concludes that psychopathological variables are the possible predisposing factors of specific addictive behaviours such as problematic gaming, gambling, and compulsive shopping (25). This link can be bi-directional since addictive behaviours could in turn intensify personal characteristics such as mental health risks (25). The pathway model of problematic mobile phone use also suggests that mental health problems such as anxiety and loneliness can be predicting factors for problematic use of mobile phones (11).

Social media has been defined as the “web 2.0 capabilities of producing, sharing, and collaborating on content online” including a wide range of social applications such as weblogs, social networking sites (mainly for connecting people) and virtual games (8), p. 2. The overuse of social media can become addictive and cause negative consequences in life (7, 8). Many cross-sectional studies found that PSMU was associated with mental health risks such as anxiety and depression (3, 4, 14). A recent systematic review based on 1,747 papers identified a strong and bi-directional relationship between problematic social media use and anxiety or depression (4). For the Chinese context, a systematic review by Hussain et al. (3) further revealed that most studies identified the link between PSMU and depression, but the effect sizes between PSMU and anxiety were larger. It is therefore important to further explore the relationships between PSMU and different types of mental health risks besides anxiety and depression. Hussain et al. (3) observed that more studies, especially longitudinal studies, on social media use and psychopathological variables among Asian groups are needed since Asian social media users were found to show more social media use disorder symptoms than their Western counterparts (3, 19, 26).

### Longitudinal studies

The longitudinal studies that have investigated the relationship between different types of problematic internet use (e.g., smartphone use, gaming, and social media use) and mental health risks and/or well-being, have obtained diverse results (6, 12, 13, 16–18, 27–33). In a three-wave longitudinal study among Chinese undergraduates, problematic smartphone use was found to be predicted by earlier stressful life events and mediated by mental health problems (33). A large-scale longitudinal study among 7,434 Chinese undergraduates revealed that psychosocial factors such as depressive symptoms, social anxiety, academic stress, and loneliness were the risk factors for problematic smartphone use (12). It thus seems that mental health issues were the key antecedents for problematic internet use, particularly smartphone use.

However, studies also revealed that the relationship between problematic internet use and mental health can be bi-directional or in a reversed direction (problematic internet use first, mental health issues later). In a three-wave longitudinal study, Teng et al. (29) revealed that earlier internet gaming disorder negatively predicted subsequent psychosocial well-being (self-esteem, life satisfaction, and social support) but not vice versa. A two-wave study among Chinese adolescents found a bi-directional relationship between internet addiction and depression but not anxiety. Earlier depression predicted subsequent internet addiction and vice versa (13). Similarly, Li et al. (18) found a bi-directional relationship between social media addiction and depression among 4,237 Chinese adolescents in a two-wave study. Therefore, the association between problematic internet use and mental health risks appears to be complex, which might be different in terms of different types of internet use (e.g., gaming, social media) and kinds of mental health risks (e.g., anxiety and depression). There is a need for more longitudinal research studies that investigate specific problematic online behaviours (e.g., PSMU) and associations with mental health risks.

Many recent longitudinal studies have investigated the relationship between PSMU and mental health risks among different groups (16, 34–37). Several longitudinal studies conducted among school children identified the link between PSMU or social media addiction and psychological distress (34, 35, 37). For example, Chen et al. (35) found that increased PSMU were associated with greater psychological distress (anxiety, depression and stress) among young school children. Besides, several studies obtained similar findings among university students using longitudinal designs [e.g., (16, 36)].

However, additional longitudinal studies on such links (PSMU and mental health risks) among first-year undergraduate students in China are still further needed to better understand the complex mechanisms of social media use and how they may facilitate the development of problematic use. Some studies have explored internet gaming disorder or problematic smartphone use among freshmen in university [e.g., (12, 29)], a focus on PSMU is needed to better understand the phenomenon. Given the potential impact of the sharp change from high school to university on Chinese first-year undergraduates’ technology use and well-being (19), it is necessary to explore the link between PSMU and mental health among first-year college students in education transition in China. This age-group tends to be technologically adept and could be prone to maladaptive behaviours, thus it is important to investigate this population.

### The present study

Given that the relationship between problematic internet use (either generalized or specific use) and mental health risks remains unclear, and that most studies have adopted cross-sectional designs, it is important to investigate the relationship between PSMU (one of the most popular specific online behaviours) and multiple mental health risks utilizing a longitudinal study design. In the Chinese context, previous studies reported that Chinese first-year undergraduate students had difficulties in adapting to a “more relaxed university life” after stressful high school years (19). It is thus necessary and reasonable to focus on these freshmen in their educational transition from high school to university. Given the cross-sectional nature of previous research and a lack of studies examining social media use amongst first-year Chinese students, the present study utilised a three-wave longitudinal study design to investigate PSMU and associations with the mental health risks of depression, anxiety, stress and loneliness amongst first-year Chinese students. The aforementioned variables were examined at three time points during the first year of university study. The study research hypotheses were as follows.

*H1*: The levels of problematic social media use severity will increase during the first year of university studies.

*H2*: Problematic social media use severity in earlier times will positively and significantly predict subsequent mental health risks.

*H2a*: Problematic social media use severity at time 1 (T1) will positively and significantly predict mental health risks at time 2 (T2).

*H2b*: Problematic social media use severity at T2 will positively and significantly predict mental health risks at time 3 (T3).

*H3*: Mental health risks in earlier times will positively and significantly predict subsequent problematic social media use severity.

*H3a*: Mental health risks at T1 will positively and significantly predict problematic social media use severity at T2.

*H3b*: Mental health risks at T2 will positively and significantly predict problematic social media use severity at T3.

**Figure 1:**
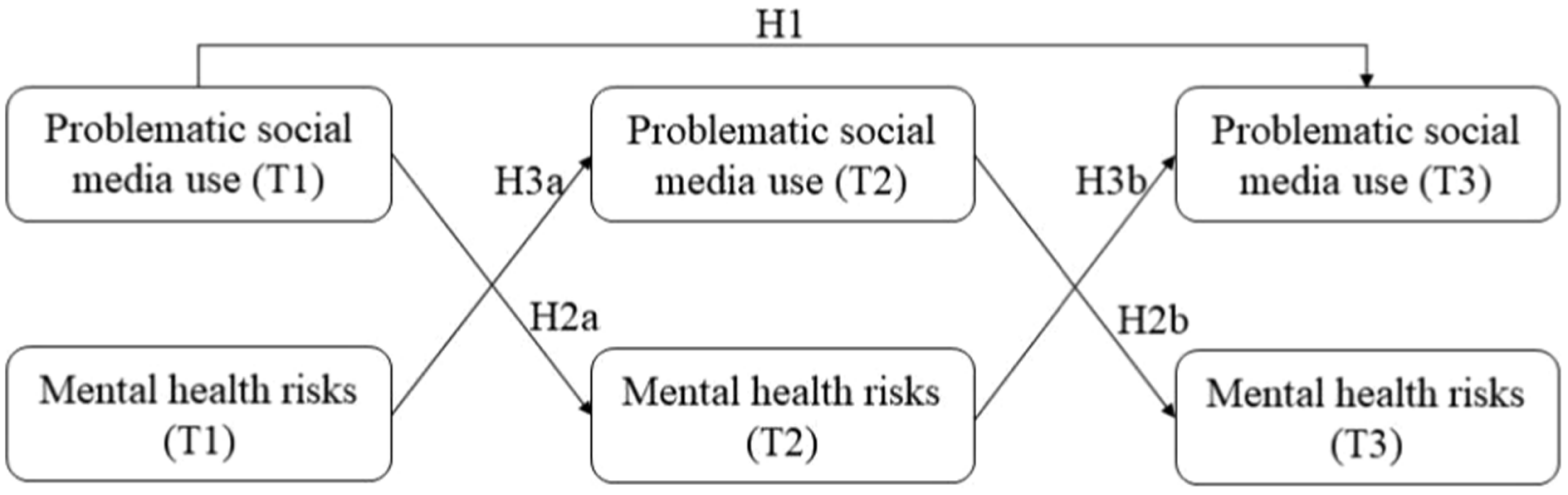
The hypothesized model.

The hypothesized model for the longitudinal associations between problematic social media use and mental health risks are shown in Figure 1.

## Methods

### Design

The study utilised a three-wave longitudinal design, data was gathered at three time points. The variables under investigation were PSMU, depression, anxiety, stress, and loneliness.

### Participants

A total of 685 Chinese first-year undergraduates completed all the three waves of surveys. The average age was 19.12 years (SD = 0.92). There were 221 males and 464 females.

### Measures

The surveys consisted of demographic questions and the following psychological scales which were translated into Mandarin.

#### Bergen social media addiction scale

The Bergen social media addiction scale [BSMAS (22)] based on the component model (38) was used to measure PSMU severity. In this study, the validated Chinese version of the BSMAS (39) was adopted. The scale consists of 6 items that assess symptoms of addiction over the past 12 months, participants rate all items on a 5-point Likert scale (where 1 = very rarely, 2 = rarely, 3 = sometimes, 4 = often, 5 = very often). Example items include “How often during the last year have you tried to cut down on the use of social media without success?” and “How often during the last year have you used social media in order to forget about personal problems?.” Total scores are obtained by summing participant ratings of each item, with higher scores indicating higher PSMU severity. To identify PSMU in the current study, a cut-off point of 24 was used. This was based on previous social media research among Chinese adolescent (40). The Cronbach’s alpha of the scale in this study was 0.85 (T1), 0.85 (T2), and 0.86 (T3), respectively.

#### Depression anxiety stress scale

The 21-item depression anxiety stress scale (DASS-21) developed by Lovibond and Lovibond (41) and validated in Chinese by Gong et al. (42) was used to measure symptoms of depression, anxiety, and stress. The DASS-21 includes 37-item subscales for depression, anxiety, and stress, respectively, rated on a 4-point Likert scale ranging from 0 (did not apply to me) to 3 (applied to me very much, or most of the time). Example items are: “I felt that life was meaningless” (depression), “I felt scared without any good reason” (anxiety), “I felt that I was rather touchy” (stress). Scores are summed with high scores indicating elevated depression, anxiety, and stress symptoms. The Cronbach’s alpha of the sub-scales in this study were all above 0.70: depression (T1: 0.84, T2: 0.85, T3: 0.86), anxiety (T1: 0.79, T2: 0.82, T3: 0.82) and stress (T1: 0.78, T2: 0.82, T3: 0.82).

#### Loneliness scale

Loneliness was measured using the 6-item Chinese version of UCLA loneliness scale [ULS-6 (43)] developed from the 20-item UCLA loneliness scale (44). Participants rate items using a 4-point Likert scale ranging from 1 (never) to 4 (always). Example items include “People are around me but not with me,” “I feel left out,” and “I lack companionship.” Loneliness is measured by calculating the average score across the 6 items. Higher scores indicate higher levels of loneliness. The Cronbach’s alpha in this study was 0.85 (T1), 0.86 (T2), and 0.87 (T3).

### Procedure

The ethics committee of the first author’s university approved the study. The study was conducted in three universities in South China and three waves of identical online surveys were distributed to the same participants. Data were collected from October 2021 to May 2022, with four-month intervals between each wave. The baseline survey (time 1, T1) recruited 822 first-year undergraduate students and 699 of them completed the second survey at time 2 (T2). In the third wave at time 3 (T3), 685 participants remained in this study and completed the third survey with 3 participants’ age missing.

All participants were presented with consent forms at the beginning of each survey, informed consent was obtained from all participants, and study participation was voluntary. As the questionnaires were anonymous, all the participants were asked to provide the last 6 digits of their phone number for the purpose of data matching. After completing all the three rounds of surveys, the participants received 20 Chinese RMB for study participation.

### Analytic strategy

Descriptive statistics and Pearson’s product-moment correlation coefficients were calculated using IBM SPSS version 23. One-way repeated measures ANOVA was conducted to compare the differences in the variables across time. Cross-lagged analysis was conducted using structural equation modelling in AMOS version 24. We tested the link between the sum scores (besides the separate scores) of mental health risks and PSMU because we intended to explore whether overall and specific mental health risks are differentially associated with PSMU. The evaluation of the model fit was done with standard criteria: comparative fit indices (CFI/TLI; values >0.90 indicated an acceptable fit with the data), and root mean square error of approximation (RMSEA; values <0.08 indicated a good fit with the data) (45, 46). Additionally, the *χ*^2^ test was used to check the data derivation of the defined model. However, before analysing the structural equation model all relevant variables were checked to see if they correlated with each other (47).

## Results

### Descriptive statistics and correlations

Table 1 shows the descriptive statistics and Pearson’s correlation coefficients of the main study variables in the three waves. There was a rise in PSMU severity over the three waves of the study. The percentages of the participants who scored higher than 24 on the BSMAS were 2.5% (T1), 3.6% (T2), 4.7% (T3) respectively. PSMU severity at T1, T2 and T3 were significantly and positively correlated with each other (*p* < 0.01). In each wave, PSMU severity was significantly and positively correlated with all the mental health risks variables (depression, anxiety, stress, and loneliness) (*p* < 0.01). To assess whether PSMU, loneliness, depression, stress, and anxiety differed over time, one-way repeated measures ANOVA were conducted. For PSMU, the main effect of time was significant, *F* (2, 1368) = 9.292, *p* < 0.001, *η*^2^ = 0.013. Specifically, the level of PSMU severity at T3 was significantly higher than T1. For loneliness, the main effect of time was significant but with a small effect size, *F* (2, 1368) = 4.915，*p =* 0.008, *η*^2^ = 0.007, which indicated that the level of loneliness at T3 was significantly lower than T1 and T2. For depression, the main effect of time was also significant, *F* (2, 1368) = 14.894, *p* < 0.001, *η*^2^ = 0.021, which indicated that the level of depression at T1 was significantly lower than T2 and T3. Similarly, participants’ perceived stress at T3 was significantly higher than T2, *F* (2, 1368) = 3.664, *p* = 0.026, *η*^2^ = 0.005. For anxiety, the main effect of time was not significant, *F* (2, 1368) = 2.272, *p* > 0.05. The skewness and kurtosis values show that all scores were normally distributed (see Table 1). The values for skewness and kurtosis between −2 and +2 are considered acceptable in order to prove normal univariate distribution (48). Hair et al. (49) and Byrne (50) argued that data is considered to be normal if skewness is between −2 to +2 and kurtosis is between −7 to +7.

**Table 1:** Correlations between problematic social media use (PSMU) and mental health risks at three time points.

Variables	1	2	3	4	5	6	7	8	9	10	11	12	13	14	15
1. PSMU (T1)	—														
2. PSMU (T2)	0.53^**^	—													
3. PSMU (T3)	0.54^**^	0.58^**^	—												
4. Depression (T1)	0.22^**^	0.22^**^	0.15^**^	—											
5. Depression (T2)	0.18^**^	0.34^**^	0.21^**^	0.61^**^	—										
6. Depression (T3)	0.21^**^	0.29^**^	0.34^**^	0.59^**^	0.70^**^	—									
7. Anxiety (T1)	0.28^**^	0.28^**^	0.23^**^	0.71^**^	0.49^**^	0.46^**^	—								
8. Anxiety (T2)	0.21^**^	0.38^**^	0.24^**^	0.50^**^	0.76^**^	0.58^**^	0.61^**^	—							
9. Anxiety (T3)	0.20^**^	0.31^**^	0.36^**^	0.46^**^	0.58^**^	0.77^**^	0.57^**^	0.68^**^	—						
10. Stress (T1)	0.33^**^	0.29^**^	0.24^**^	0.68^**^	0.49^**^	0.47^**^	0.78^**^	0.57^**^	0.52^**^	—					
11. Stress (T2)	0.23^**^	0.43^**^	0.27^**^	0.48^**^	0.74^**^	0.55^**^	0.55^**^	0.82^**^	0.61^**^	0.61^**^	—				
12. Stress (T3)	0.24^**^	0.34^**^	0.40^**^	0.47^**^	0.56^**^	0.75^**^	0.51^**^	0.60^**^	0.83^**^	0.56^**^	0.65^**^	—			
13. Loneliness (T1)	0.24^**^	0.24^**^	0.20^**^	0.50^**^	0.35^**^	0.35^**^	0.49^**^	0.36^**^	0.35^**^	0.50^**^	0.36^**^	0.36^**^	—		
14. Loneliness (T2)	0.18^**^	0.33^**^	0.18^**^	0.43^**^	0.54^**^	0.44^**^	0.40^**^	0.49^**^	0.41^**^	0.40^**^	0.52^**^	0.43^**^	0.58^**^	—	
15. Loneliness (T3)	0.16^**^	0.31^**^	0.28^**^	0.41^**^	0.48^**^	0.55^**^	0.39^**^	0.44^**^	0.52^**^	0.41^**^	0.46^**^	0.55^**^	0.57^**^	0.66^**^	—
M	15.93	16.33	16.68	4.07	4.59	4.73	4.84	4.60	4.65	6.18	5.87	6.18	13.22	13.19	12.86
SD	4.89	4.77	4.78	3.71	3.90	3.98	3.46	3.57	3.61	3.72	3.92	3.90	3.73	3.80	3.71
Skewness	0.00	−0.07	0.05	1.33	1.05	1.19	1.00	1.03	1.13	0.57	0.64	0.58	−0.01	−0.01	0.09
Kurtosis	−0.40	−0.38	−0.07	1.86	0.86	1.55	1.34	1.05	1.69	0.26	0.19	0.29	−0.32	−0.40	−0.18

^**^p < 0.01, *N* = 685, M = mean values, SD = standard deviation.

### Attrition analyses

We conducted attrition analysis following the approaches in previous studies (51). Our attrition analyses did not show any significant differences between participants who dropped out (16.7%) and those who took part in all three surveys when considering all the variables: gender, *χ*^2^(1) = 1.25, *p* = 0.26; age, *t*(817) = 0.58, *p* = 0.56; PSMU, *t*(820) = −0.25, *p* = 0.80; loneliness, *t*(820) = 1.64, *p* = 0.10; stress, *t*(820) = 1.00, *p* = 0.32; depression, *t*(820) = 1.24, *p* = 0.21; anxiety, *t*(820) = 1.05, *p* = 0.30. Therefore, there was no need to conduct data imputation for further analysis.

### Structural equation modelling

**Figure 2:**
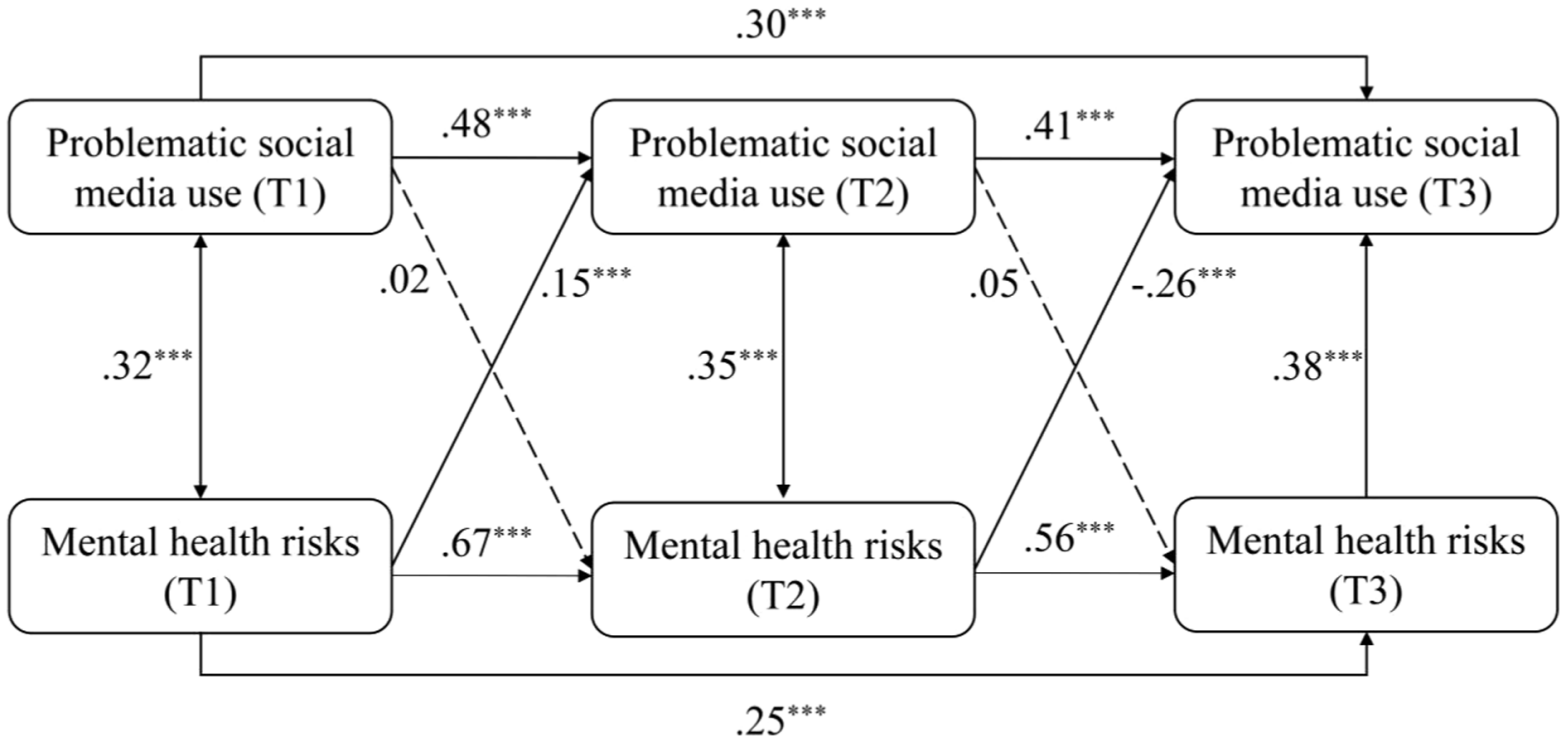
Model 1: the cross-lagged model of PSMU and mental health risks. ^*^p < 0.05, ^**^p < 0.01, ^***^p < 0.001, *N* = 685.

Table 2 shows the cross-lagged model fits for the longitudinal relationship between PSMU severity and mental health risks (anxiety, depression, stress, and loneliness). The model fit indices were acceptable at good levels and indicate that all the models fitted the data well. As shown in Figure 2, the model including PSMU scores and the total scores of mental health risks obtained a good model fit (*χ*^2^/*df* = 2.17, CFI = 0.999, TLI = 0.991, RMSEA = 0.041). PSMU in earlier times did not significantly predict mental health risks in subsequent times from T1 to T2 and from T2 to T3. Mental health risks positively and significantly predicted PSMU from T1 to T2 (*β* = 0.15, *p* < 0.001) but not from T2 to T3 (*β* = −0.26, *p* < 0.001). However, mental health risks positively and significantly predicted PSMU in T3 (*β* = 0.38, *p* < 0.001). Furthermore, PSMU in earlier times positively and significantly predicted subsequent PSMU, mental health risks in earlier times positively and significantly predicted subsequent mental health risks.

**Table 2:** Model fits of the cross-lagged models for PSMU and mental health risks (anxiety, depression, stress and loneliness).

Model	Mental health risks in the model	*χ*^2^/*df*	CFI	TLI	RMSEA
1	Total score	2.17	0.999	0.991	0.041
2	Depression	1.28	0.999	0.997	0.020
3	Anxiety	2.89	0.998	0.982	0.053
4	Stress	1.62	0.999	0.994	0.030
5	Loneliness	1.79	0.999	0.992	0.034

The total score was computed by summing up the scores of anxiety, depression, stress and loneliness.

**Figure 3:**
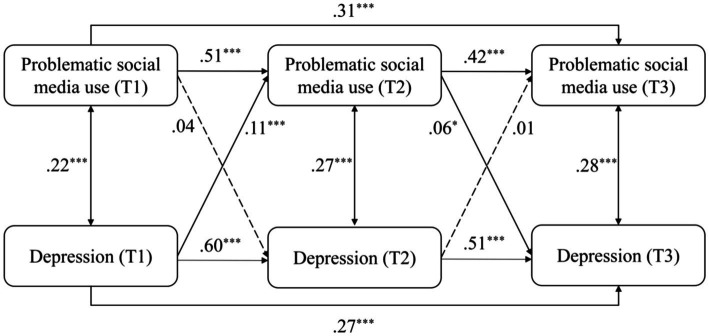
Model 2: the cross-lagged model of PSMU and depression.

**Figure 4:**
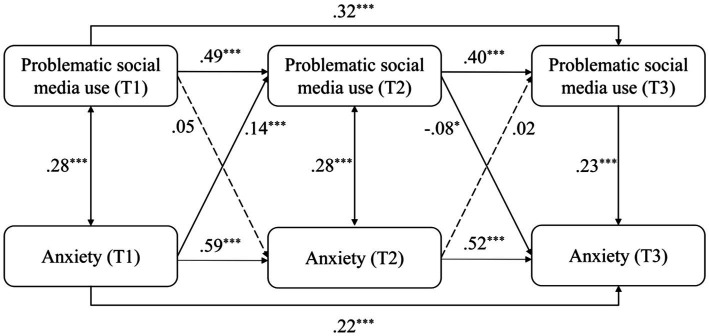
Model 3: the cross-lagged model of PSMU and anxiety.

**Figure 5:**
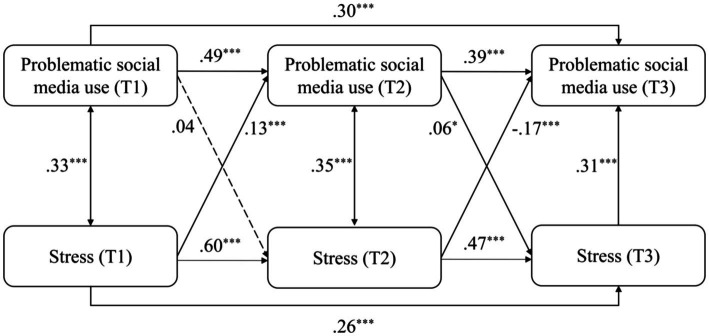
Model 4: the cross-lagged model of PSMU and stress.

**Figure 6:**
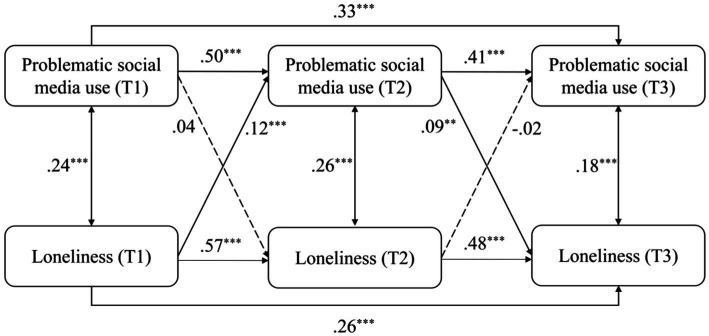
Model 5: the cross-lagged model of PSMU and loneliness.

When replacing the total scores of mental health risks by the scores of depression, anxiety, stress, and loneliness, the models were similarly structured and obtained good model fits (see Table 2). The models were differently modified according to modification indices to reach good model fits. For the model of depression (see Figure 3), depression positively and significantly predicted PSMU from T1 to T2 (*β* = 0.11, *p* < 0.001), and PSMU significantly predicted depression from T2 to T3 (*β* = 0.06, *p* < 0.05). For the model of anxiety (see Figure 4), anxiety positively and significantly predicted PSMU from T1 to T2 (*β* = 0.14, *p* < 0.001). For the model of stress (see Figure 5), stress positively and significantly predicted PSMU from T1 to T2 (*β* = 0.13, *p* < 0.001) but not from T2 to T3 (*β* = −0.17, *p* < 0.001). PSMU positively predicted stress from T2 to T3 (*β* = 0.06, *p* < 0.05). For the model of loneliness (see Figure 6), loneliness at T1 significantly predicted PSMU at T2 (*β* = 0.12, *p* < 0.001), and PSMU at T2 could predict the level of loneliness at T3 (*β* = 0.09, *p* < 0.01).

## Discussion

### Summary of the findings

The present study examined the relationship between PSMU and mental health risks (depression, anxiety, stress, and loneliness) using a three-wave longitudinal design. The results showed an increase in PSMU severity over time among first-year Chinese undergraduate students. PSMU severity at T3 was significantly higher than PSMU severity at T1, which supported H1. Overall, the correlational analysis revealed positive longitudinal associations between PSMU severity and mental health risks. However, in the cross-lagged models, this relationship was different over the two intervals. Earlier PSMU severity did not significantly predict subsequent mental health risks which reject H2a and H2b. Mental health risks significantly and positively predicted PSMU severity from T1 to T2 but not from T2 to T3, which support H3a and reject H3b. In T3, mental health risks positively and significantly predicted PSMU severity. It is possible that there was a suppressor effect in the model (52), which indicates that mental health risks in the same research waves were more powerful predictors of PSMU severity compared with earlier mental health risks. This can be seen from the correlational analysis that most of the contemporaneous coefficients between PSMU severity and mental health risks were larger. Another reason for the negative path from T2 mental health risks to T3 PSMU might be the role of stress which negatively predicted PSMU from T2 to T3 (see Figure 5). It possibly indicates that those first-year undergraduates who faced increasing levels of stress in the second half of the year (as shown in our ANOVA) had limited free time for social media and perceived lower PSMU subsequently. The longitudinal associations were different when replacing the total scores of mental health risks by depression, anxiety, stress, and loneliness separately (see models 2 to 5). Bi-directional longitudinal relationships between PSMU severity and the four variables were identified but not consistent across all the three waves of studies.

### Theoretical and practical implications

The present study supports the theories of specific internet use disorder and PSMU. The I-PACE model suggest that psychopathological variables can be the predicting factors for addictive behaviours (25). In line with this, the present study proved that PSMU severity was consistently positively correlated with mental health risks in all the three waves of the study. Mental health risks in T1 significantly predicted PSMU severity in T2, which also proves that existing mental health problems could act as the risk factors for subsequent PSMU. These findings are similar to the findings of previous longitudinal studies that reported problematic internet use can be predicted by psychopathological variables such as anxiety and loneliness (12, 33). The present study seems to prove that mental health risks were key risk factors for PSMU severity.

The relationship between PSMU severity and mental health risks was also reported as bi-directional in several previous longitudinal studies (18) and by the I-PACE model, which was not consistently identified in all models in the present study. Instead, PSMU severity measured at the same waves were stronger predictors of mental health risks, while the effects of earlier PSMU were weak. The longitudinal link between PSMU and mental health risks can be different between different intervals in this study. For example, depression significantly predicted PSMU severity from T1 to T2 but not vice versa, while this link was only significant from PSMU to depression from T2 to T3. The possible reason for this might be the different research context of the participants, for example, different term times or the changing university experiences. Thus, the bi-directional link between PSMU and mental health needs further investigation.

Besides, the model of the sum scores of mental health risks was different from the models for separate variables in the current study. Such difference is possibly caused by the difference between anxiety, depression, stress and loneliness, as they represent different aspects of mental health. Previous studies tested the links between PSMU and single mental health risks separately [e.g., (6, 33)] or overall psychological distress (using the sum scores or latent variables) [e.g., (35, 36)]. Chen et al. (36) used the sum scores of anxiety and depression to represent the overall psychological distress and found that initial PSMU predicted the growth of psychological distress. However, little is known about the different results between the sum scores of mental health risks and separated ones with PSMU. Studies testing the relationship between PSMU and separate mental health risks did not further explore such links using the sum scores [e.g., (6)]. Our findings indicate that it is not easy to simply conclude that social media is good or bad for people’s general mental health or overall well-being. In other words, the association between PSMU and overall well-being or mental health conditions might not represent the potential specific mental health problems related to social media use. Future studies in this topic need to test different types of mental health risks separately and be aware of their differences.

The present study enhances our understanding of the prevalence of PSMU among first-year undergraduates in China. Previous studies found that Chinese first-year undergraduates were more likely to use smartphones and the internet problematically than students from other countries since they were not ready for a more relaxed university lifestyle compared with their strictly managed high school lives (19). However, without longitudinal studies, it cannot be confirmed whether the students’ problematic internet use increase or decrease during their education transition, though they might have difficulty in adapting to a different learning and living environment. Previous longitudinal studies reported different results. Teng et al. (29) found that Chinese first-year undergraduates had increased levels of internet gaming disorder over the three waves of study. However, Wang et al. (12) reported that the junior (first and second-year) undergraduates’ problematic smartphone use decreased over 18 months. Though, the mixed sample of first and second-year students might affect the results, the trend of problematic internet use remains unclear with limited research evidence. In this context, the present study observed an increased level of PSMU severity over the freshmen’s first year at university. Using the same cut-off point, Luo et al. (40) reported that the 12 months prevalence of social media addiction among Chinese adolescents was 3.5%. The results of the present study were similar, the first-year university students who scored above the cut-off point were 2.5% (T1), 3.6% (T2), and 4.7% (T3). A clear trend of increased PSMU severity indicates that more attention should be paid to the first-year undergraduates’ problems of dealing with online applications such as social media. Such finding also suggest that early interventions are necessary (in the first term of studies) to counteract PSMU and mental health issues. For example, programmes for managing social media usage and getting used to university life.

After 8 months’ of adaption to university, the participants reported higher depression and stress but lower loneliness, which clearly shows that different types of mental health risks should be considered differently. The results from the cross-lagged models showed that the links between PSMU and different types of mental health issues were not the same. This is in line with previous studies which identified that the longitudinal effect of internet addiction on subsequent anxiety and depression were different (13). Similarly, a systematic review by Elhai et al. (53) concluded that depression was the most consistent and powerful psychopathological variable (consistent but with medium effect sizes), and correlated with problematic smartphone use compared with anxiety (consistent but with small effect sizes) and stress (less consistent with small to medium effect sizes). It is therefore important for future studies to focus on specific mental health issues when investigating problematic internet use and psychopathology.

### Limitations and future directions

There are several limitations to the present study. The risk of providing socially desirable answers to survey questions is one limitation of this study, though all the scales obtained good reliabilities with Cronbach’s alpha values larger than 0.70 in all three waves of the study. Furthermore, only self-perceived scores for PSMU and mental health risks were obtained which may not fully reflect the students’ diverse mental health conditions. Qualitative data analysis or sentiment analysis for their narrative expressions are needed besides quantitative data analysis. The sample size of 685 were not representative enough compared with previous longitudinal studies, though the present study focused on a special and under-researched group of first-year undergraduates. Thus, the results might not be generalised to other senior undergraduate students who have already adapted to their university lives much better.

Future studies could adopt different designs such as mixed-methods studies and longitudinal qualitative studies. For example, future studies could investigate the first-year undergraduates’ narrative descriptions or their social media posts to better understand mental health conditions. Furthermore, longitudinal studies with larger sample sizes or more waves of surveys can be implemented to explore the link between PSMU and mental health. Besides, systematic reviews are needed to investigate the relationships between PSMU and multiple mental health risks and to compare the effect sizes of PSMU on several different types of mental health issues. Future longitudinal studies could also adopt alternative data analysis methods such as growth modelling to explore the relationship between PSMU and mental health with particular focus on the growth of the variables.

## Conclusion

The present study used a three-wave longitudinal design to investigate the relationship between PSMU severity and mental health risks among Chinese first-year university students. The results showed that PSMU was positively correlated with mental health risks, and the effect sizes were larger for this link in the same waves. Cross-lagged models revealed that earlier mental health risks positively predicted subsequent PSMU severity, and bi-directional associations were identified but not consistent in all models over all waves of studies. The presents study indicates that Chinese first-year undergraduates’ levels of PSMU can increase during their adaptation year in university. The present study serves to highlight the importance of implementing longitudinal research designs to better understand PSMU. The findings can inform prevention and intervention programmes (e.g., behaviour focused and youth development programmes) to tackle PSMU among university students.

## Data availability statement

The original contributions presented in the study are included in the article/supplementary materials, further inquiries can be directed to the corresponding author.

## Ethics statement

The studies involving humans were approved by Ethics Committee of School of Education at Soochow University. The studies were conducted in accordance with the local legislation and institutional requirements. The participants provided their written informed consent to participate in this study.

## Author contributions

WZ, ZYY, and ZHY: concept and design, acquisition, analysis, interpretation of data, and statistical analysis. ZYY, ZHY, WZ, and ZH: drafting of the manuscript and critical revision of the manuscript. All authors contributed to the article and approved the submitted version.

## Funding

The present study was funded by the National Social Science Fund of China under grant number 22CSH077; the Humanity and Social Science Youth Foundation of the Ministry of Education of China under grant number 21YJCZH200; Social Science Youth Foundation of Jiangsu Province under grant number 21XWC005; High level personnel (Shuang chuang) project of Jiangsu Province under grant number JSSCBS20210698; Postgraduate Research & Practice Innovation Program of Jiangsu Province under grant number SJCX23-1654.

## Publisher’s note

All claims expressed in this article are solely those of the authors and do not necessarily represent those of their affiliated organizations, or those of the publisher, the editors and the reviewers. Any product that may be evaluated in this article, or claim that may be made by its manufacturer, is not guaranteed or endorsed by the publisher.

## Conflict of interest

The authors declare that the research was conducted in the absence of any commercial or financial relationships that could be construed as a potential conflict of interest.
